# Supplementary material for: Portable Prussian Blue-Based Sensor for Bacterial Detection in Urine
Source: Sensors (Basel). 2022 Dec 30;23(1):388. doi: 10.3390/s23010388 (PMC9823789; doi:10.3390/s23010388)
Supplement: Supplementary file 1 [file sensors-23-00388-s001.zip › sensors-2090676-supplementary.pdf]

Portable Prussian Blue-Based Sensor for Bacterial Detection in Urine

Carolin Psotta,[1,2], Vivek Chaturvedi,[1,3], Juan F. Gonzalez-Martinez,[1,3], Javier Sotres [1,3], and Magnus Falk [1,3] *

[1] Department of Biomedical Science, Faculty of Health and Society, Malmö University, Malmö 20506, Sweden

[2] Aptusens AB, 293 94 Kyrkhult, Sweden

[3] Biofilms-Research Center for Biointerfaces, Malmö University, Malmö 20506, Sweden

*corresponding author

Supplementary Material

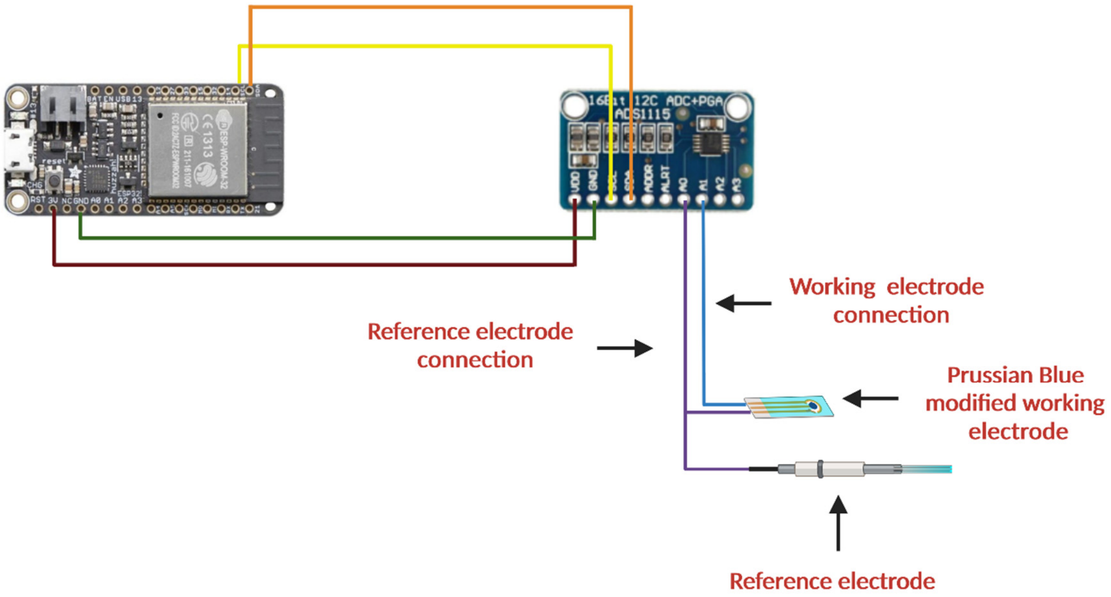

Supporting Figure S1: The Adafruit HUZZAH32 (left) connected to the ADS1115 digital to analog converter. The ADS1115 also connects the working electrode and the reference electrode.

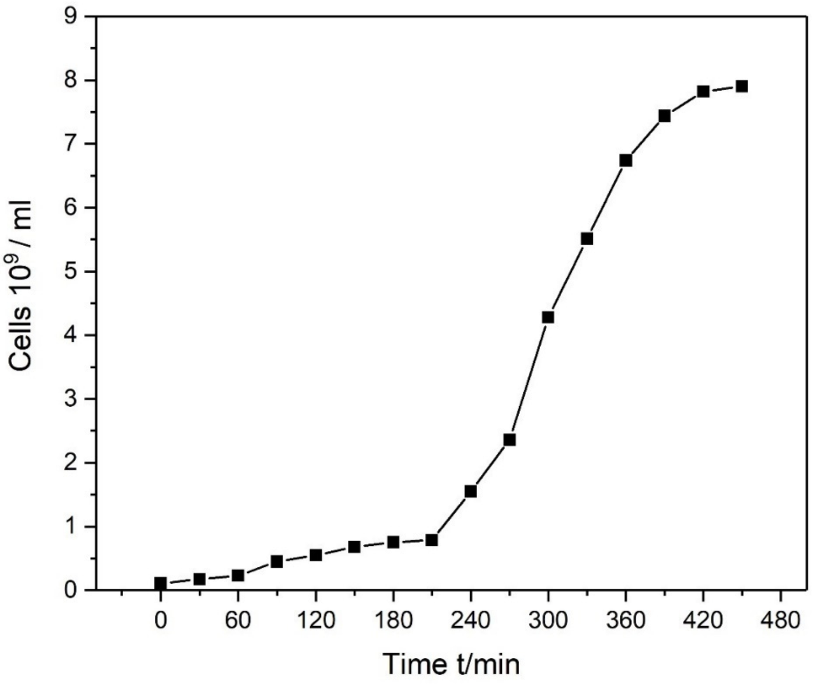

Supporting Figure S2: Growth curve of *E.coli* in AUM

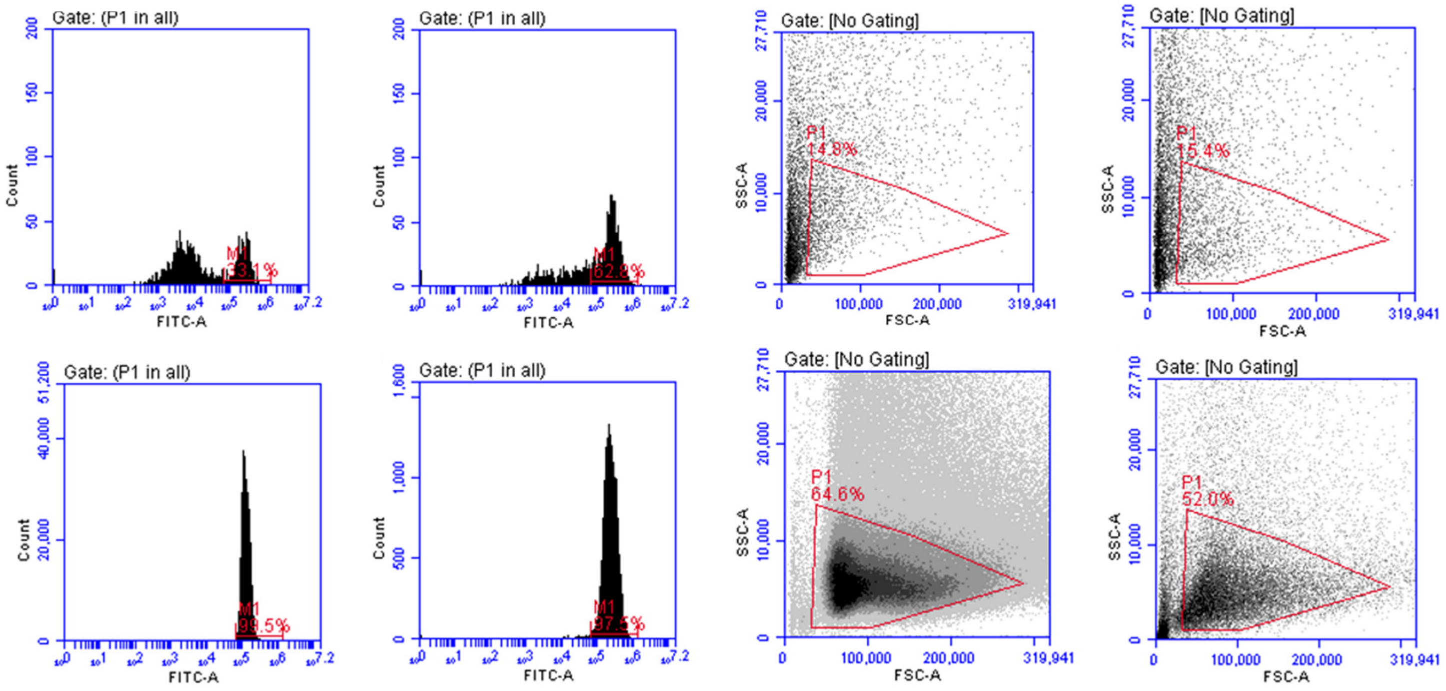

(**a**) (**b**)

Supporting Figure S3: (a) Flow cytometry: FITC-A vs. cell count of the analyzed aliquots after incubation at 0, 2, 4, and 6 h (clockwise); **(b)** Flow cytometry: FSC-A vs. SSC-A of the analyzed aliquots after incubation at 0, 2, 4, and 6 h (clockwise)

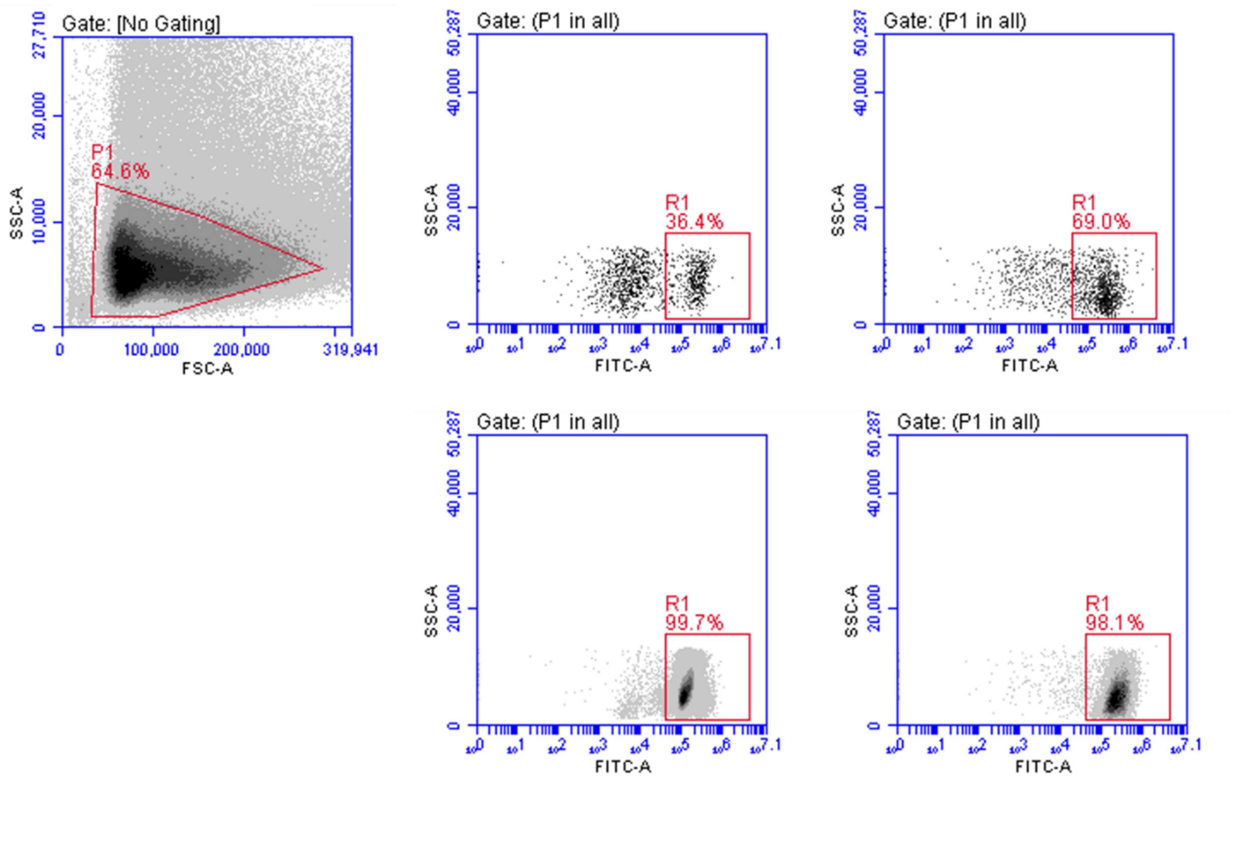

Supporting Figure S4: Flow cytometer results by displaying FITC-A vs. SSC-A (Fluorescein isothiocyanate vs. side scatter). Chosen cut cutoff point was set at 8000 (background noise reduction). P1 gate was set in an unstained sample containing bacteria in AUM, and the gate was unchanged for the different time points. Separate scatter plot with the related R1 gate (see Figure 6b; 0 h = 36.4; 2 h = 69.0 %; 4 h = 98.1 %; 6 h = 99.7 %, clockwise).

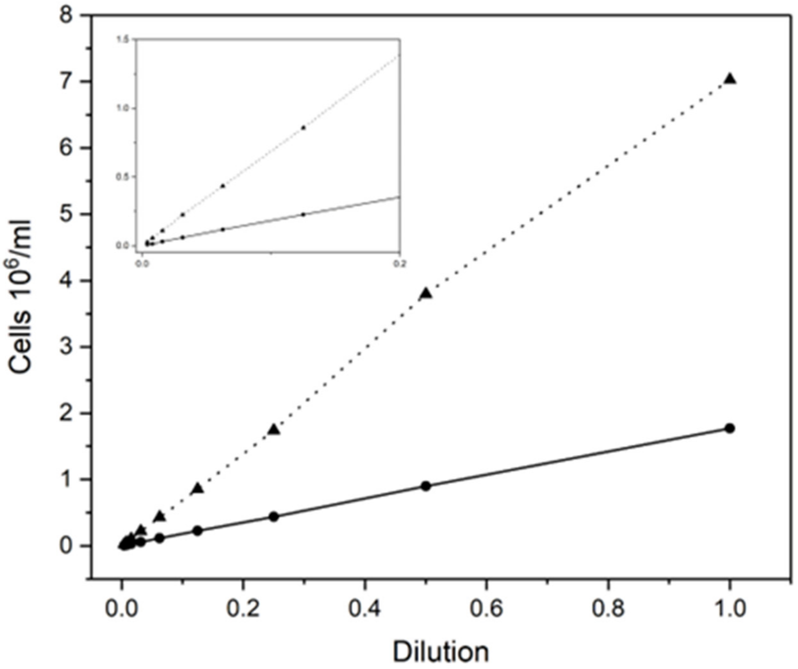

Supporting Figure S5: Flow cytometry: testing different dilutions (1, 0.5 (1:2), 0.25 (1:4), 0.125 (1:8), 0.0625 (1:16), 0.03125 (1:32), 0.01563 (1:64), 0.00781 (1:128), 0.00391 (1:256) in AUM (solid circle) and authentic urine (dotted triangle)

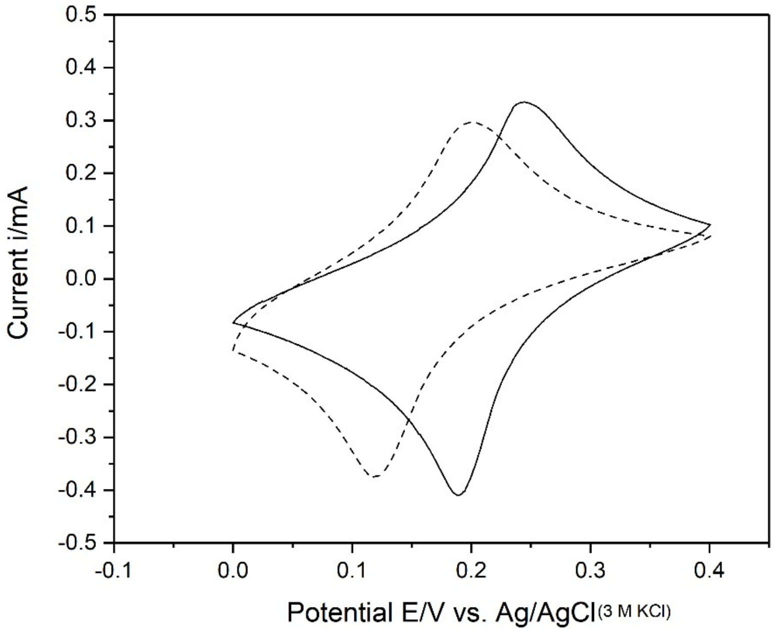

Supporting Figure S6: CVs of PrBl-modified SPEs in 0.1 M HCl and two different KCl concentrations, recorded for a deposition time of 40s (solid = 1 M KCl; dashed = 0.1 M KCl), recorded at a sweep rate of 50 mVs^{-1}

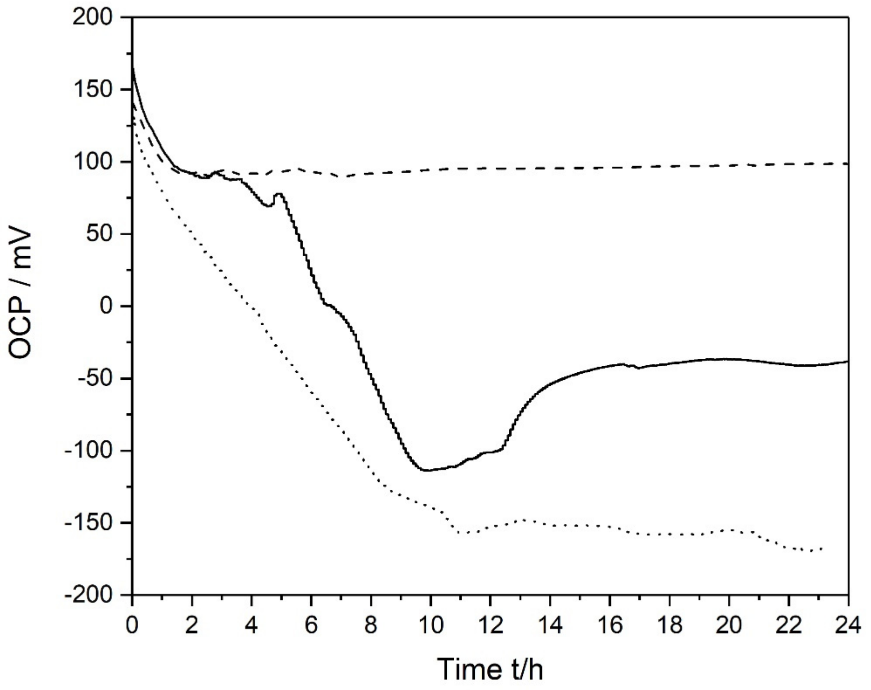

Supporting Figure S7: Measurement in AUM for 24 h (solid = PrBl-modified + 2 µl bacterial suspension; dotted = PrBl-modified + 200 µl bacterial suspension; dashed = PrBl-modified + no bacteria)

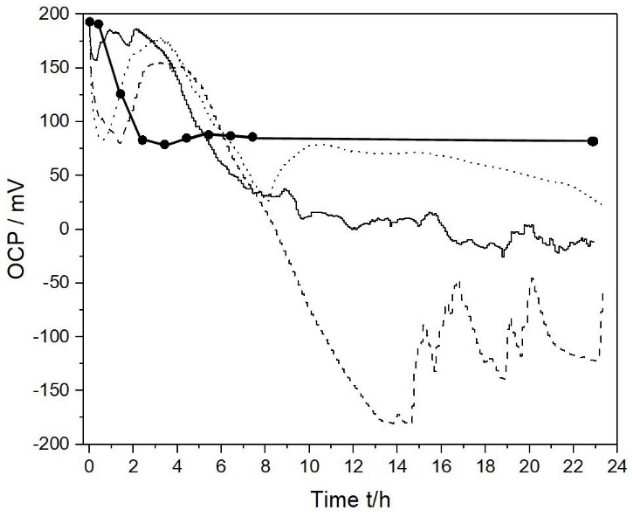

(**a**)

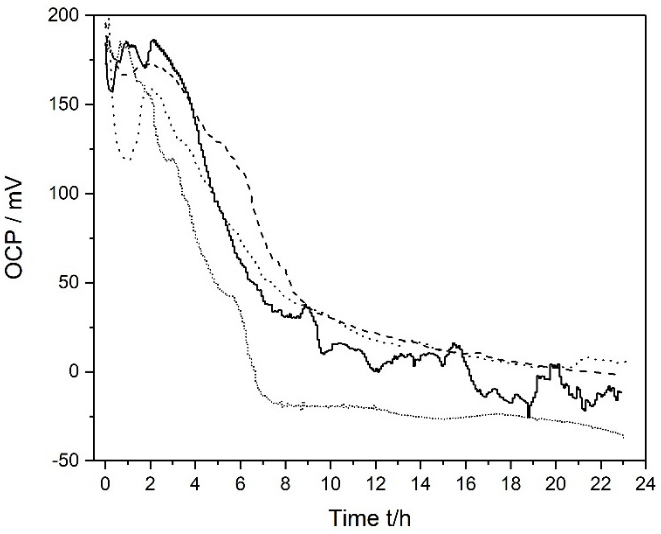

(**b**)

Supporting Figure S8: (a) Measurement in human urine for 24 h (solid = PrBl-modified + 200 µl bacterial suspension; dotted = PrBl-modified + 100 µl bacterial suspension; dashed = PrBl-modified + 10 µl bacterial suspension; solid with large dots = PrBl-modified + no bacteria), **(b)** 200 µl of bacteria in human urine at different urine sample collection times and for two different volunteers, volunteer one and afternoon collection (solid), volunteer one and morning collection (dashed), volunteer two and afternoon collection (short dots), volunteer two and morning collection (dotted).
